# Ovarian Cancer Stem Cells: Role in Metastasis and Opportunity for Therapeutic Targeting

**DOI:** 10.3390/cancers11070934

**Published:** 2019-07-03

**Authors:** Xingyue Zong, Kenneth P. Nephew

**Affiliations:** 1 Medical Sciences, Indiana University School of Medicine, Bloomington, IN 47405, USA; 2 Department of Cellular and Integrative Physiology and Obstetrics and Gynecology, Indiana University School of Medicine, Indianapolis, IN 46202, USA; 3 Indiana University Simon Cancer Center, Indianapolis, IN 46202, USA

**Keywords:** cancer stem cells, metastasis, ovarian cancer

## Abstract

Ovarian cancer (OC) is a heterogeneous disease usually diagnosed at a late stage. Cancer stem cells (CSCs) that exist within the bulk tumor survive first-line chemotherapy and contribute to resistant disease with metastasis. Understanding the key features of CSC biology provides valuable opportunities to develop OCSC-directed therapeutics, which will eventually improve the clinical outcomes of patients. Although significant developments have occurred since OCSCs were first described, the involvement of CSCs in ovarian tumor metastasis is not fully understood. Here, we discuss putative CSC markers and the fundamental role of CSCs in facilitating tumor dissemination in OC. Additionally, we focus on promising CSC-targeting strategies in preclinical and clinical studies of OC and discuss potential challenges in CSC research.

## 1. Introduction

Ovarian cancer (OC) is the leading cause of death from gynecologic malignancies, with approximately 22,240 new cases reported in the United States annually [1]. It is estimated that 75% of OC patients present with disseminated disease within the peritoneal cavity at the time of initial diagnosis, rendering metastasis a prevalent issue in OC treatment. Currently, the standard therapy for OC comprises debulking surgery followed by taxane- and platinum-based chemotherapy. Although this regimen is initially effective, up to 80% of women with advanced stage ovarian high-grade serous carcinoma (HGSOC) experience recurrence with metastatic disease, and the five-year survival rate is approximately 30% [1,2,3]. There is an urgent need to better understand the mechanism of tumor spread because platinum-resistant metastasis is one of the most challenging issues in OC.

Recent data have pointed to the persistence of quiescent ovarian cancer stem cells (OCSCs) not eliminated by chemotherapy that are able to regenerate tumors as the main contributor to tumor relapse and metastasis. Understanding the molecular and biological features of OCSCs may allow for effective targeting and eradicating of these cells, resulting in potential tumor remission. 

In this review, we focus on how cancer stem cells (CSCs) are defined and isolated in OC, discuss the driver role of OCSCs in both passive and hematogenous metastasis models, and summarize promising agents targeting OCSCs in preclinical and clinical settings. Although anti-CSCs strategies remain in the early stage of research, targeting OCSCs represents an extraordinary opportunity to improve the chance of progression-free survival among OC patients.

## 2. Definition and Identification of OCSCs

The CSC hypothesis proposes that a subpopulation of neoplastic cells exist within a tumor which have an increased ability to self-renew, generate diverse cells in the tumor mass, and sustain tumorigenesis [4,5,6]. CSCs are also largely referred to as tumor-initiating cells. The first evidence for CSCs came in a 1994 study, which proved that one CD34+/CD38-cell from human acute myeloid leukemia (AML) could reinitiate leukemia in mice [7]. Since 1994, CSCs have been under intensive investigation in breast, brain, colon, lung, and prostate [8,9,10,11]. Over a decade ago, Bapat et al. first reported the existence of CSCs or progenitor cells in OC patient ascites, suggesting that cells from a single clone can resemble original tumor [12]. Subsequently, we were the first group to identify and characterized OCSCs from HGSOC patient samples [13]. 

The CSC hypothesis applies to OC for several reasons. For example, both ovarian surface epithelium (OSE) and fallopian tube epithelium (FTE) express multiple stem cell markers, indicating both are able to give rise to OCSCs. Furthermore, recent studies demonstrated a cancer-prone stem cell niche at the hilum of the OSE in mice, characterized by cells expressing numerous stemness markers (ALDH1, LGR5, LEF1, CD133, CK6B) [14]; five stem cell markers (NANOG, SFRP1, LHX9, ALDH1A1, and ALDH1A2) were detected in both OSE and FTE [15]. Specifically, these markers are detected more often in the distal FTE, which also represents the putative precursor of HGSOC [14,15]. Notably, CSC theory provides an explanation for frequent disease recurrence with massive peritoneal tumor nodules despite the initial response to treatment [16,17,18]. In addition, the majority of patients present with peritoneal ascites, which may represent the desired environment for survival and enrichment of OCSCs [19].

Subsequent studies revealed biological features of CSCs in cancer progression, particularly with respect to chemoresistance [20,21,22] and metastasis [23,24,25]. Moreover, the standard platinum-based treatment of OC leaves residual tumors with enhanced CSC-like traits, resulting in an enhanced metastatic potential [26,27]. In addition, a recent study suggested that PARP inhibitors, a FDA-approved monotherapy for recurrent OC patients, increased both the OCSC population and ability to repair DNA [28]. Moreover, a study in triple-negative breast cancer showed that RAD51-mediated resistance of CSCs to PARP inhibition, regardless of BRCA1 status [29]. Thus, the CSC theory generates an exciting new area in cancer research that shows great promise to fully overcome OC recurrence and metastasis. In this regard, the identification and isolation of OCSCs is a crucial prerequisite. The gold standard of CSC definition is based on the serial transplantation ability in vivo. However, putative OCSC markers have been considered a valuable tool to track CSCs and predict tumor progressions; however, the accuracy of marker-based CSC identification is still debatable, and there is no consensus on a universal marker. Functional assays, such as in vivo limiting dilution assay, in vitro spheroid formation assays, and aldefluor assays, are conducted as complementary evidence to confirm cancer stemness. In addition, expression of stemness-related genes, for example OCT4, NANOG, SOX2, is routinely analyzed to suggest cell plasticity at molecular level. Here, markers of OCSCs are reviewed below in the order first reported in the literature.

CD44 is a surface transmembrane glycoprotein that acts as a receptor for different microenvironmental cues and affects gene expression levels related to cellular differentiation and cell-matrix adhesion. CD44 is one of the most common CSC surface markers, used either alone or in combination with other putative markers, to identify CSCs in OC and other cancers [13,30,31,32]. For example, injection of a hundred CD44+CD117+ cells isolated from OC patient tumors was capable of propagating the original tumor; however, CD44−CD117− cells were nontumorigenic [13]. Alvero et al. identified CD44+ cells in primary and metastatic tumors as well as in malignant ascites [33]. In addition, CD44+ cells possess a distinctive genetic profile regarding tumorigenicity, chemoresistance, constitutive NFκB activity, and has the potential to promote a pro-inflammatory tumor microenvironment [33]. Despite the function of CD44 as a stem cell biomarker, contradictory findings suggest that CD44 fails to function as a prognostic factor in OC [34,35].

CD117, commonly known as c-kit, is a receptor tyrosine kinase that is involved in multiple cell signaling related to maintaining fundamental cellular functions such as cell survival, metabolism, and differentiation. Overactivation of CD117 has been reported across different cancers. In OC, high expression of CD117 has correlated with poor disease-free survival rate and potential peritoneal metastasis [36,37]. Luo et al. showed that purified CD117+ cells from OC could repopulate the original tumor with high heterogeneity, suggesting CD117+ cells possess self-renewal and differentiation ability [38]. Furthermore, as a receptor tyrosine kinase, CD117 has exhibited the ability to drive chemoresistance and has tumor-initiating capacity via activation of Wnt/β-catenin-ATP-binding cassette G2 signaling in OC cell lines [39]. As mentioned above, CD117 has been used in combination with CD44 to identify OCSCs [13].

CD24 is a small cell surface marker that is highly expressed in a variety of cancers [40,41], including in approximately 70% of primary tumors obtained from 174 OC patients [42]. Gao et al. demonstrated that CD24+ cells exhibit quiescent and a more chemoresistant phenotype when compared with CD24− cells; 5000 CD24+ cells initiated tumorigenesis in vivo while the same amount of CS24− cells did not [43]. In accordance with this finding, Butgos-Ojeda et al. showed that CD24+ cells versus CD24− cells had greater tumor-forming potential in murine OC model with APC, PTEN, and TP53 deletion [44]. Beyond the role of a CSC marker, CD24 is also functionally associated with cell adhesion, contributing to the attachment of tumor cells to fibronectin or collagen during metastasis [41]. CD24 is capable of inducing epithelial to mesenchymal transition (EMT) via PI3K/AKT and MAPK pathways, supporting the possibility that CD24 a significant metastatic progression marker for poor clinical outcome in OC [42,45].

Aldehyde dehydrogenase (ALDH) is a family of enzymes, including 19 isoforms, that promote the oxidation of aldehyde substrates to their corresponding carboxylic acids [46,47]. ALDH+ cells have exhibited improved DNA repair and increased drug efflux transporters in OC, suggesting a functional role in mediating drug resistance [48,49]. Because ALDH+ cells demonstrate different aspects of CSC features, numerous studies have chosen ALDH level to define OCSCs [50,51,52,53]. Clinically, a higher percentage of ALDH+ cells was significantly associated with poor outcome in serous OC patients (n = 439, *p* = 0.0036) [54]. Although ALDH has been viewed as a robust OCSC marker, little is known about the roles of different ALDH family isoforms and how they contribute to cancer stemness individually and cooperatively, which increases the complexity of designing targeting inhibitors [55]. A recent study demonstrated a supporting role of ALDH1A2 in maintaining OCSC phenotypes, which is comparable with the ALDH1A1 [56]. New knowledge of other isoforms will facilitate improved understanding of ALDH functions in OCSCs.

CD133, a glycosylated transmembrane protein, is also frequently expressed in cancers and has prognostic value in OC. Numerous signaling pathways mediated by CD133 can modulate cancer stemness and metastasis [57,58]. Baba et al. reported that CD133+ OC cells generate both CD133+ and CD133− populations; however, CD133− cells could only divide symmetrically. Moreover, CD133+ cells showed increased chemoresistance. CD133+ cells could also form more aggressive tumor xenografts when compared with CD133− subpopulation [59]. Similarly, CD133+ cells isolated from human primary ovarian tumor displayed higher tumorigenic capacity when injected into NOD/SCID mice and were capable of recapitulating the original heterogeneous tumor [60].

## 3. Roles of OCSCs in Metastasis Models

**Figure 1:**
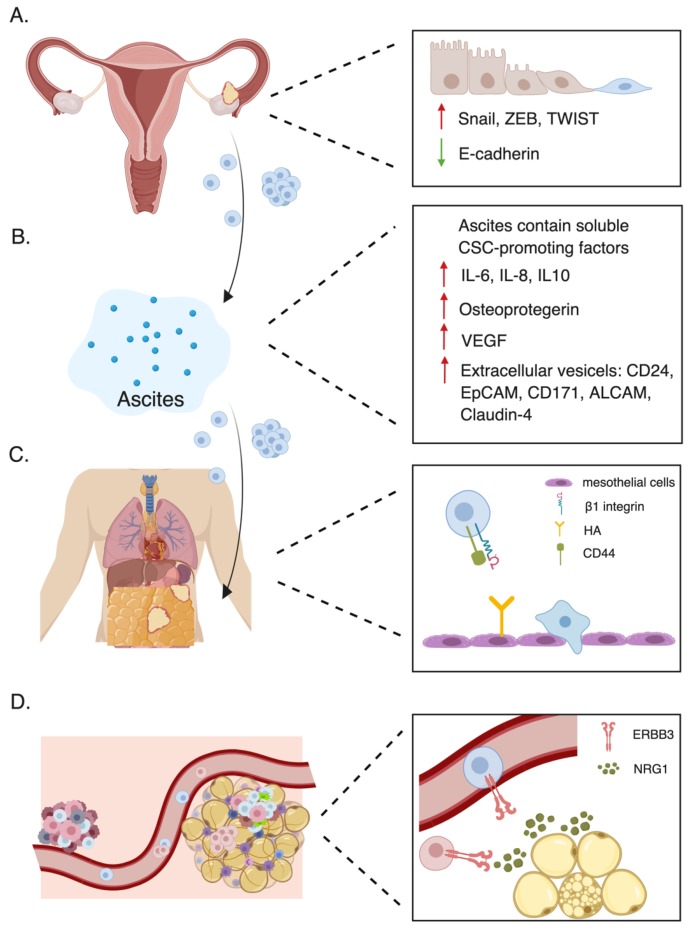
The involvement of OCSCs in passive tumor dissemination (**A**–**C**) and in hematogenous metastasis (**D**). (**A**) The cancer cells at primary site may undergo EMT, gain stem cell properties, and disperse into ascites as either single cell or multicellular spheroids. (**B**) Ascites provides floating cells with a CSC-promoting microenvironment. (**C**) Adhesion to mesothelium is facilitated by CD44 and β1 integrin heterodimer on the surface of floating cells. (**D**) Activated ErbB3/NRG1 axis promotes hematogenous metastasis to omentum.

The majority of OC-related death are due to chemoresistant metastasis [3]. Unlike other tumors, in which hematogenous metastasis is recognized as a primary pattern of disease spread, OC metastasis follows a unique route of dissemination. OC normally metastasizes within the peritoneal cavity to other pelvic and peritoneal organs via circulation of ascites [3,61]. Although this passive dissemination is viewed as the dominant mechanism of metastasis, recent studies have pointed out the existence of an active mode of metastasis in which OC cells enter the blood circulation and reseed to secondary sites [62]. According to data from 1481 OC patients, the most common distant metastatic location is the liver, followed by distant lymph nodes, lung, bone, and brain [63]. In both metastasis models, OCSCs played a fundamental role in facilitating the metastatic cascade, as seen in Figure 1.

### 3.1. Passive Dissemination

Unlike the vast majority of tumors, the cell of origin of epithelial OC remains controversial. The traditional theory indicates that OC derives from ovarian surface epithelium and subsequently develops into different histological subtypes of OC. Some believe that OC tumorigenesis initiates from Müllerian type cysts located in paratubal and paraovarian regions. However, the most compelling studies suggest that OC does not originate from the ovary but derives from the fallopian tube [64,65,66,67,68]. Nonetheless, classic OC metastasis route begins when OC cells lose cell–cell contact and detach from the primary tumor [69].

To overcome adhesion to neighboring cells, some OC cells may undergo EMT and loosen cell contacts, which may also contribute to the acquisition of stem cell characteristics. Once the EMT program is triggered by extracellular stimuli, the transcriptional factors associated with EMT (EMT-TFs) act cooperatively to drive cellular reprogramming [70]. Well-known EMT-TFs, including Snail, ZEB, and TWIST families, are also key regulators of CSC biology [71], orchestrating gene expression changes via promoter activation or repression; such EMT-TFs eventually confer CSC properties to epithelial-state cells, such as via specific CSC marker expression and activation of CSC-associated signaling [72,73]. This acquired plasticity is coupled to dedifferentiation of tumor cells, which increases malignant potential of cells and expands intratumoral diversity [74,75,76]. However, the role of EMT in transforming cancer cells to CSCs and contributing to drug resistance remains an open question. In OC, greater expression of E-cadherin is observed in primary tumors when compared with detached tumor cells in peritoneal fluid [77]. Moreover, Snail and Slug have been reported to mediate self-renewal programs during EMT, leading to resistance to p53-mediated apoptosis in OC [78]. Consequently, these observations suggest that EMT activation permits OC cells to detach from the primary site and potentially redefines the stemness status of differentiated of OC cells. However, it is debatable whether these stem-like tumor cells are truly dedifferentiated OCSCs with full tumorigenic capacity and whether targeting EMT could be an effective avenue to eliminate OCSCs. Overall, fundamental knowledge regarding the origin of OCSCs is needed, such as malignant transformation of normal stem cells or dedifferentiation from cancer cells, and this remains an active area of investigation.

Next, these stem-like tumor cells disperse into ascites as either single cell or multicellular spheroids throughout the peritoneal cavity [3]. Ascites, the excess fluid in the abdominal cavity, is detected in more than one third of OC patients at initial diagnosis [79]. Multiple studies have shown ascites to be a rich source of OCSCs [80,81,82,83]. This non-adherent microenvironment is lethal to adherent tumors cells, and only cells with mesenchymal features can tolerate the anoikis stress and survive. Ascites contains a variety of tumor-promoting soluble factors that contributes to CSC enrichment, such as interleukin (IL)-6, IL-8, IL-10, osteoprotegerin, vascular endothelial growth factor (VEGF), and extracellular vesicles (EVs) [84,85,86,87]. We recently reported that IL-6 regulates stemness features of CSCs by activating STAT3 signaling and enhancing ALDH1A1 expression [88]. In addition, several studies have emphasized the importance of EVs in promoting cancer progression, which adds another level of complexity to study the microenvironment of ascites. Beyond the traditional role of a biomarker, EVs represent a novel mode of communication between cells by transfer cytosolic proteins, lipids, RNA, and DNA in ascites. This directly regulates cellular functions of both tumor cells and host cells in a paracrine fashion, eventually resulting in the enrichment of OCSCs and tumor migration [89,90,91,92]. For example, Runz and colleagues identified CD24 and EpCAM as cargo proteins of exosomes in cell lines and malignant ascites, which are both stemness and prognostic markers of OC [93]. Other molecules carried by EVs reported in OC include L1 adhesion molecule (CD171), activated leukocyte cell adhesion molecule (ALCAM), CD44 and claudin-4 [94,95,96,97,98]. Given the variety of potential factors contributing to CSC maintenance, ascites is considered to promote the acquisition of the stem cell state.

Floating OC cells travel along with the ascites, with the movement of respiratory force, before settling onto the new sites. Adhesion to mesothelium, the lining of peritoneal cavity, is the first step of implantation. This step is facilitated by CD44 and β1 integrin heterodimers on the surface of floating OC cells, which are ligands for hyaluronic acid (HA) and the extracellular matrix molecules on mesothelial cells [99,100,101,102]. Intriguingly, mesothelial cells facilitate cancer stemness properties in spheroids of OC cells, including increasing CD44 expression, suggesting a positive feedback loop in adhesion step between mesothelium and floating OC cells [103]. In addition, CD133 regulated by ARID3B has been reported to promote mesothelial attachment [104]. Once these floating OC cells initiate the implantation process, usually at abdominal peritoneum or omentum, the surrounding stromal cells at metastatic sites are stimulated to create a favorable microenvironment for the implant growth [105,106]. Moreover, supportive factors secreted in the microenvironment contribute to CSC self-renewal and conversion from non-CSCs to CSCs [107,108]. For example, IL-17, when released by macrophages, could promote the self-renewal ability of OCSCs by the nuclear factor (NF)-κB and p38 mitogen-activated protein kinases (MAPK) signaling pathway [109]; Leukemia inhibitory factor (LIF) and IL-6 produced by OC-associated mesenchymal stem cells promote OCSC via STAT3 signaling pathway [110]. In addition, adipose tissue, the main component of omentum, can promote cancer stemness and metastasis via a highly orchestrated network [111,112,113]. Overall, different steps in passive dissemination require cells with stem-like properties to survive; simultaneously, the process of OC spread also creates favorable conditions for CSC enrichment.

### 3.2. Hematogenous Metastasis

Although passive dissemination is believed to be the major mechanism of OC metastasis for decades, an alternative pattern has been recently reported. Several studies have detected circulating OC cells in patient blood, highlighting a hematogenous route of OC dissemination [114,115,116]. Circulating tumor cells (CTCs) have long been considered to share characteristics with CSCs [117,118,119]. For example, molecular characterization of single CTCs from OC patients was found to be positive for stemness markers (CD44, ALDH1A1, NANOG, OCT4) and EMT markers (N-cadherin, Vimentin, Snai2, CD117, CD146) [116]. A recent study revealed that CTCs self-renew, express CSC markers, and have multilineage differentiation and tumorigenic ability [120]. On the basis of the prevalent “seed and soil” theory, Pradeep et al. demonstrated the non-random pattern of hematogenous dissemination of ERBB3+ circulating OC cells, which preferably metastasize to omentum with high neuregulin1(NRG1) level [62]; ERBB3 was previously shown to enable glioblastoma CSC proliferation [121]. Although CTCs display some hallmarks of CSCs, whether there is interconversion between these two populations or whether they are essentially the same population remains unclear. Furthermore, the connection between OCSCs and metastatic dormancy remains poorly understood. Collectively, the incidence of CTCs has shed light on the hematogenous mechanism of OC metastasis; however, the extent that CTCs resemble CSCs requires additional investigation.

## 4. Therapeutic Strategies

**Figure 2:**
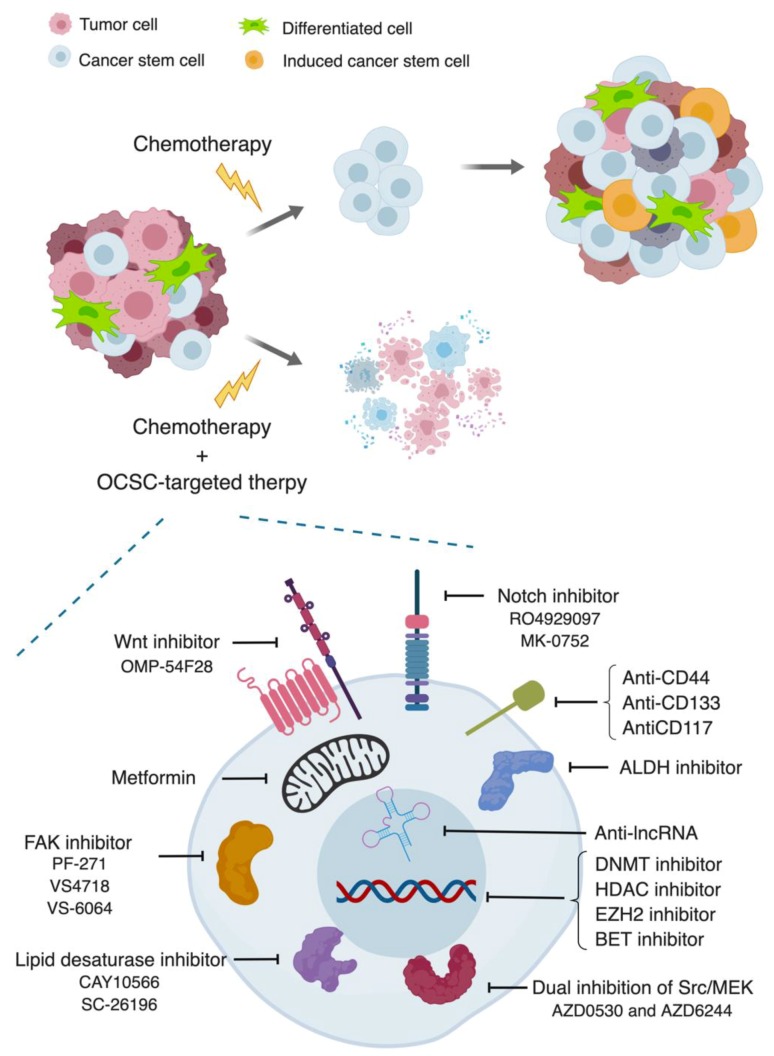
Schematic representation of OCSCs driving recurrent tumors and targeting strategies. Standard chemotherapy eliminates bulk tumor but not OCSC population. The residual tumor is enriched in OCSC population post-chemotherapy, which generates diverse cell population and drives a more aggressive disease. However, combining conventional chemotherapy with selected anti-OCSC therapeutics can ultimately contribute to potential tumor remission.

Given the critical roles of OCSCs in mediating metastasis, it is crucial to eradicate CSCs using targeted therapies. Compelling studies have described the distinct features of OCSCs, which has been and may continue to be the main rational for CSC-directed drug design. Current strategies mainly involve targeting OCSC markers, epigenetic features, stem cell signaling, metabolic traits and microenvironment. Promising results have been observed in preclinical models and clinic settings, either alone or in combination with traditional cytotoxic drugs. The successful disruption of cancer stemness can eventually slow cancer progression or even cure cancer. Here, we summarize the anti-CSCs therapeutics that have been specifically evaluated in OC, as seen in Figure 2.

### 4.1. Stemness Markers

Identification and isolation of OCSCs reliant on unique cell surface/intracellular markers is a common practice in research. In fact, the majority of these markers are functionally important to CSC biology. Therefore, strategies have been introduced both to recognize CSC-specific antigen and then to kill them with cytotoxic conjugates but also to inhibit biological function of stemness markers, especially ALDH in OC. In addition, for surface marker targeting, novel approaches have been created, including antibody-drug conjugates, chimeric antigen receptor T (CAR-T) cells and bispecific antibodies [122].

#### 4.1.1. Anti-CD44

Over the past decade, various monoclonal antibodies [123,124] and small interfering RNAs [125] against CD44 isoforms, carried by novel drug delivery systems, have been developed in OC. Although these regimens demonstrate attractive anti-tumor efficacy, there are no anti-CD44 therapeutics available in the clinic for OC patients, potentially owing to the substantial side effects on normal stem cell which expresses high level of CD44 [126]. In addition to its role as a CSC surface marker, CD44 also serves as a ligand of HA. As noted above, this binding directs the attachment of OC cells to mesothelium, independent of CSC properties, which results in oncogenic activation of different signaling pathways associated with metastasis [127,128]. Consequently, therapeutics that interfere with HA-CD44 interaction have been extensively explored [32,129,130]. For example, treatment with a small HA oligosaccharide inhibited the growth of CD133+ OC cells in vivo via inhibiting association of CD44 with the receptor tyrosine kinases (RTK), monocarboxylate (lactate) transporters (MCT), and ATP-binding cassette (ABC) family multidrug transporters [129].

#### 4.1.2. Anti-CD133 

dCD133KDEL, a monoclonal antibody to a CD133 fusion protein that recognizes a non-glycosylated region of CD133, has been developed as a CSC-directed therapeutic. Although CD133-positive cells are only a minority of the population of cells in an ovarian tumor, this anti-CD133 toxin dramatically suppresses the growth of OC in vitro and OC metastasis in vivo in a human OC mouse xenograft model [131]. In addition, a chimeric antigen receptor (CAR)-based immunotherapeutic approach targeting CD133-positive cells successfully eradicated of OCSCs from OC cell lines and primary ascites harvests [132].

#### 4.1.3. Anti-CD117 

As a receptor tyrosine kinase, CD117 represents a valuable druggable target. Imatinib mesylate (Gleevec, STI571) is a competitive inhibitor with potent activity against platelet-derived growth factor receptor (PDGFR) and CD117. Although Imatinib mesylate showed efficacy against OC cell survival in vitro [133], minimal single-agent activity was seen in primary or recurrent OC patients in Phase II clinical trials [134,135]. The reason for this is not clear; however, in malignant glioma cells, it was shown that inhibition of PDGFR resulted in unexpected activation of ERK and subsequently PI3K/AKT, perhaps limiting the use of Imatinib mesylate as a single agent [136].

#### 4.1.4. ALDH Inhibitor

Several novel ALDH inhibitors have been recently evaluated in OCSC [137,138]. Recently, Chefetz et al. identified that a pan-ALDH1A family inhibitor 673A preferentially depletes OCSCs by inducing necroptosis, which is highly synergistic with cisplatin in reducing tumor initiation capacity in vivo [139]. Another specific ALDH1A1 inhibitor CM37 eliminated OCSC via increasing DNA damage [140].

### 4.2. Epigenetic Therapies

Analogous to normal stem cells where epigenetic regulators suppresses lineage differentiation, various studies have showed that CSC maintenance requires elaborate reprogramming of the epigenome [141,142,143,144,145,146,147,148]. Aberrant epigenetic alterations, including chromatin remodeling and DNA methylation changes, are common features of OC and other cancers, which can cause partial or even complete loss of epigenetic constraints in cancer cells [149,150,151,152,153,154]. This gain of plasticity may allow cells to lose typical epithelial phenotype and become invasive and chemoresistant. Epigenetic drugs are recognized as differentiation therapy in which CSC are induced to undergo differentiation from quiescent/pluripotent state to differentiated state through the activation of differentiation-associated signaling cascades and re-expression of tumor suppressor genes. Hence, CSCs can be converted to more differentiated cancer cells, making them susceptible to conventional cytotoxic drugs. With manageable off-target effects, epigenetic drugs hold great promise to serve as broad reprogrammers of OCSCs.

#### 4.2.1. DNA Methyltransferase Inhibitor (DNMTi) 

Dysregulation of DNA methylation results in transcriptional silencing of differentiaeion-related genes as well as tumor suppressor genes. Over the last decade, various DNMTis have been investigated and translated into the clinic, demonstrating significant clinical efficacy when combined with platinum [155,156,157,158,159]. Low dose SGI-110 (Guadecitabine), a next-generation DNMTi, successfully differentiated ALDH+ OCSCs, reduced their stemness properties and eventually re-sensitized OCSCs to platinum-based therapy [160], demonstrating proof of concept for epigenetic targeting of OCSCs.

#### 4.2.2. Histone Deacetylase Inhibitor (HDACi) 

HDACi function as differentiation inducers in CSC-targeted therapy by inhibiting gene expression related to CSC maintenance, such as g HIF-1α, Stat3, Notch1, β-catenin, NF-κB, and c-Jun [161]. HDACis have been extensively explored in both preclinical and phase I/II/III clinical settings; such studies have revealed synergistic or additive efficacy when combined with other anticancer agents [162,163,164,165]. Two HDAC family members, HDAC1 and HDAC7, were found specifically overexpressed in OCSCs when compared with the non-stemness counterparts. Overexpressing HDAC7 alone was sufficient to increase tumor initiating capacity in vivo, indicating that HDAC7 is an essential epigenetic regulator to maintain CSC phenotype [144]. Moreover, selective HDAC1 and HDAC7 inhibitors preferentially target CSCs and inhibit xenograft tumor growth in OC [144]. In addition, Tang et al. invented a drug screening pipeline to search for EMT reversal molecules and discovered HDACi to be a promising candidate capable of restoring epithelial differentiation in OC [166]. Furthermore, functional studies showed that HDACi could promote anoikis and impair spheroid formation capacity in OC cells [166].

#### 4.2.3. Histone Methyltransferase Inhibitor (HMTi) 

EZH2 overexpression is widely observed in OC and other malignancies; however, it displays both tumor-promoting and tumor-suppressing features. The contradiction may be due to the fact that EZH2 can modulate a wide range of transcription programs and lineage-specifying factors conferring diverse cell fates [150]. Rizzo et al. demonstrated greater expression of EZH2 in side population of cells in comparison with its counterpart from OC ascites, suggesting EZH2 enhances OCSCs survival in OC patients after chemotherapy [81]. Additionally, EZH2 inhibited OCSC survival through miR-98 regulating pRb–E2F signaling pathway [167]. We observed that EZH2 induces enrichment of H3K27me3 at promoter loci of DAB2IP, a critical tumor suppressor, in a OCSC population, leading to enhanced survival of OCSCs and other malignant properties, including migration ability and chemoresistance (unpublished). However, Li et al. utilized genome-wide approaches to demonstrate that ALDH1A1 is directly repressed by EHZ2, indicating that EZH2 inhibition enhanced OCSC marker expression [168]. Given the complex role of EZH2 in OC, there are no ongoing clinical trials of EZH2 inhibitors specifically in OC. However, EZH2 inhibitors, either alone or in combination with other agents, hold great potential in the treatment of OC.

#### 4.2.4. Bromodomain and Extraterminal Inhibitor (BETi) 

As epigenetic readers, BET family members regulate transcription via the recognition of covalent histone modifications by their bromodomains. JQ1, a selective bromodomain 4 (BRD4) inhibitor, induced squamous differentiation, accompanied by cell growth arrest, suggesting JQ1 can be used as potential CSC differentiation therapy [169]. Using an unbiased screen system, Yokoyama et al. discovered that BET inhibitors decreased ALDH enzymatic activity and ALDH1A1 expression in OC by targeting ALDH1A1 super enhancer [170]. Furthermore, the addition of JQ1 potentiated the antitumor effects of chemotherapy, suggesting a promising strategy for CSC-directed targeting in OC [170]. Several preclinical studies reported the efficacy of combing BETis with other targeted agents, such as PI3K inhibitors, ERK inhibitors, and PARP inhibitors in OC. However, there currently are no ongoing clinical trials in OC [171]. BRD4 inhibition reveals significant antitumor effects in PDX xenografts derived from OC strains with high MYC expression, indicating that a subset of OC patients with this genetic feature may benefit from BETi [172].

#### 4.2.5. Long Non-Coding RNAs (lncRNAs) 

LncRNAs play critical roles in tumor progression through the formation of interaction complexes with DNA, RNA, and proteins [173]. HOX antisense intergenic RNA (HOTAIR) is responsible for cellular senescence via activating NF-kB signaling and increasing IL-6 secretion in platinum-resistant OC [174]. To target aberrant HOTAIR expression, Ozes et al. have designed a peptide nuclei acids (PNA)-based approach to interfere the EZH2 binding to HOTAIR, which decrease CSC population in OC cell lines and hence resensitize resistant cells to platinum treatment [175], providing proof of concept for targeting HOTAIR in OCSCs.

### 4.3. Signaling Pathways

Dysregulation of key signaling pathways is a driving force of CSC emergence and metastatic initiation. Common pathways include Wnt, Notch, and Hedgehog (Hh) signaling pathways, which are essential for cell self-renewal, differentiation, proliferation, and mesenchymal features [176]. Therefore, components of these signaling pathways are viewed as prime targets. However, these pathways are not mutually exclusive and there is substantial crosstalk among different pathways. Moreover, these pathways are not activated specifically in CSCs but also in normal tissue; they play an important role in normal stem cell maintenance, which renders efficient targeted therapies difficult to achieve. Signaling pathways supporting OCSC phenotypes have been extensively reviewed elsewhere [177,178,179]. In this review, we focus on new therapeutics in the clinic.

Wnt signaling is implicated in tumor cell dedifferentiation and stemness functions [180,181]. Ipafricept (OMP-54F28), a novel Wnt pathway inhibitor, is a first-in-class recombinant fusion protein that competes with frizzled family receptor 8 (Fzd8) for binding to Wnt ligands [182]. Preclinical studies with OMP-54F28 have demonstrated anti-CSC effect and reduced tumor growth in OC xenograft models [183]. A phase 1a study has been completed in patients with advanced solid tumors and is currently being tested in combination with standard chemotherapy in ovarian, pancreatic, and hepatocellular cancers [184,185].

Notch pathway is one of the most intensively studied putative therapeutic targets of CSCs [186,187]. γ-secretase inhibitors (GSIs) are Notch pathway inhibitors, capable of depleting OCSCs and increasing tumor sensitivity to platinum. Moreover, combination of GSI and platinum-based therapy can simultaneously eliminate OCSCs and bulk tumor by enhancing the DNA damage response and cell arrest [188]. However, a phase II study of RO4929097, a GSI, in patients with recurrent platinum-resistant epithelial OC demonstrated no evidence of objective response to single drug treatment [189]. A novel potent GSI, MK-0752, has shown promising antitumor effects in a preclinical study of OC and is currently under assessment in clinical trials in various cancers [190]. In addition, Enoticumab (REGN421), a monoclonal antibody that binds human Delta-like (Dll)4 and disrupts Notch-mediated signaling, has been examined in a phase I study. Enoticumab monotherapy is well-tolerated and four out of eight OC patients demonstrated a significant (>50%) serum CA-125 decrease [191]. 

### 4.4. Other

Focal adhesion kinase (FAK) is a protein tyrosine kinase that has been implicated in the maintenance of CSCs, potentially via activation of β-catenin pathway [192,193]. Preclinical studies have revealed that targeting FAK with small molecule inhibitor PF-271 prevents anchorage-independent cell growth in vitro and reduction of peritoneal metastasis in vivo [194]. Another FAK inhibitor, VS-4718, was shown to diminish cisplatin-resistant OCSC properties, such as reduced aldefluor activity and secondary tumor initiation frequency [195]. VS-6063, a second-generation FAK inhibitor, tested in a phase I dose-escalation study, demonstrated modest activity in advanced OC in combination with paclitaxel [196,197]. Furthermore, low Merlin expression was reported in FAK inhibitor sensitive OC lines, indicating that Merlin may be a biomarker to predict FAK inhibitor response [198].

Metformin, a standard drug for diabetes, demonstrated inhibitory effects on OC cell proliferation, spheroid formation ability of ALDH+ cells and the growth of ALDH+ cell xenografts [199]. In addition, metformin inhibited OC growth by inducing cell cycle arrest and increasing paclitaxel sensitivity in a mouse model [200]. Low-dose metformin selectively targeted CD44+CD117+ OCSCs through reversing EMT [201]. The anti-OCSC effect of metformin may be due to the modulation of energy metabolism [202]. Recent studies have recognized metformin as a novel therapeutic option of OC metastasis [203,204] and metformin plays different anti-CSC roles in multiple tumor types [205].

Lipid desaturation was recently reported to be a metabolic hallmark of OCSCs [206]. High levels of unsaturated fatty acids activates NF-κB pathway and promotes stemness phenotypes; simultaneously, activated NF-κB pathway positively regulates lipid desaturases. Moreover, lipid desaturase inhibitors, CAY10566 and SC-26196, prevented sphere formation and tumorigenesis ability. Therefore, this unique metabolic vulnerability of OCSCs represents a new target for CSC-specific therapy [206].

Src and MAPK coactivation is seen in 31% of HGSOC. Saracatinib (AZD0530), a potent Src family kinase inhibitor and Selumetinib (AZD6244), a noncompetitive MEK1/2 inhibitor, have been evaluated in OC cells, both individually and together. Dual inhibition of Src/MEK was synergistic and exhibited a marked decrease in ALDH1 level and tumor initiating ability after serial xenografting, supporting the design of an anti-CSC strategy targeting multiple pivotal signaling pathways [207].

## 5. Conclusions

Despite significant progress in understanding OCSCs, developing OCSC-targeted therapeutics remains a formidable challenge. Identification of OCSCs is a major challenge due to heterogeneity. Although putative stem cell markers are well described in established cell lines, they are not applicable to all patients due to heterogenous responses to different microenvironment cues. Notably, CSC population in one tumor is not a homogeneous group of cells. Asymmetric division, a hallmark of CSCs, enables diversification during cell division. Moreover, the dynamic co-existence of quiescent, proliferating and metastatic states of CSC may activate different signaling pathways, which results in treatment failure using a single standard treatment [208]. As a result, a thorough understanding of OCSC biology remains a top priority. In the era of precision oncology, it is expected that treatment of cancer would be customized to individual patients. For example, single cell sequencing is able to identify and characterize CSCs from bulk tumor or ascites. On the basis of this information, personalized OCSC markers and aberrant signal activation can be identified. A recent study established patient-derived OC organoid culture systems to combat heterogeneity [209]. Organoid lines captured intratumoral and intertumoral heterogeneity of the primary tumor and were xenografted for subsequent in vivo drug test [209]. This novel platform holds great promise for the personalized OCSC-based therapies.

Another barrier is that CSC-directed therapy lacks specificity from normal stem cells, resulting in a narrow therapeutic window. For example, inhibiting BET has been shown to affect intestinal stem cells, leading to GI toxicity and disruption of tissue homeostasis in multiple organs [210]. To avoid off-target effects, biological differences between CSCs and normal stem cells is needed to provide target selection. Meanwhile, considerable advancements in delivery technologies, such as using nanoparticle-mediated strategy, oncolytic viruses that replicate exclusively in cancer cells, would improve efficient intervention [211]. Together, managing adverse effects associated with targeted therapy is equally important as improving drug efficacy.

Other challenges include the design of optimal timing to give CSC-targeted treatment and development of resistance. Early intervention of CSCs, either before or co-administrated with chemotherapy, may be of most benefit to patients [122]. However, limited information is available for how long the treatment should be maintained. To address this, rigorous evaluation of anti-CSCs effects in clinic trials is necessary, including CSC frequency, chemosensitivity, stemness gene signature, and long-term tumor progression. Moreover, to minimize the rate of failure, it is critical to stratify patients according to different genetic/epigenetic background of OCSCs. Overall, an evolving understanding of CSCs would facilitate the development of CSC-targeting therapeutics and novel combinatorial treatment, contributing to long-term benefits for OC patients.
